# Human Thelaziasis: Emerging Ocular Pathogen in Nepal

**DOI:** 10.1093/ofid/ofy237

**Published:** 2018-09-15

**Authors:** Ranjit Sah, Shusila Khadka, Mahesh Adhikari, Reema Niraula, Apoorva Shah, Anadi Khatri, Suzanne Donovan

**Affiliations:** 1 Department of Microbiology, Tribhuvan University Teaching Hospital Institute of Medicine, Maharajgunj, Kathmandu, Nepal; 2 Department of Ophthalmology, BP Koirala Lions Centre for Ophthalmic Studies, Institute of Medicine, Tribhuvan University Teaching Hospital, Maharajgunj, Kathmandu, Nepal; 3 Department of Medicine (Division of infectious Diseases), Olive View-UCLA Medical Center, California; 4 Department of Medicine, Division of infectious Diseases, Medanta the Medicity, Gurugaon, Haryana, India

**Keywords:** Nepal, *Phortica variegata*, *Thelazia callipaeda*, Thelaziasis, zoonotic disease

## Abstract

Thelaziasis is an ocular arthropod-borne, zoonotic disease of the eye infecting the conjunctival sac, lacrimal duct, and lacrimal gland caused by a nematode of the genus *Thelazia*. We report the first case of human ocular thelaziasis in Nepal in a 6-month-old child from a Rukum district, Nepal. The infant presented with conjunctivitis, and his visual acuity and dilated fundal examination were normal. A total of 6 worms were removed for identification. Collected nematodes were identified based on morphological keys as Thelazia *callipaeda*. The patient’s symptoms improved after removal of the nematodes.

Thelaziasis is an ocular arthropod-borne disease of the eye infesting the conjunctival sac, lacrimal duct, and gland caused by a nematode of the genus *Thelazia* [1]. In general, patients with thelaziasis are asymptomatic or present with excessive lacrimation. The 2 *Thelazia* species associated with thelaziasis-associated disease in humans are *Thelazia callipaeda* (Oriental eye worm) and *Thelazia californiensis* (California eye worm) [2]. *Thelazia* species are transmitted by different species of Muscidae [3], which are a family of flies with worldwide distribution. However, the only confirmed vector for *T callipaeda* is the fruit fly, *Phortica variegata* (Diptera, Drosophilidae, Steganinae), which feeds on ocular secretions of their definitive hosts, including cats, dogs, horses, cattle, and humans [4].

*Thelazia* species are transmitted by secretophagous flies feeding on the animal and human eyes belonging to the Drosophilidae family [2, 5]. The first-stage larvae of the parasite are ingested by the vectors along with the conjunctival secretions of infected animals; they mature into their third larval (infective) stage in 2–3 weeks within the vector [6]. When the vector (fly) feeds in the eye of a new definitive host, including humans, the larvae emerge from the labella of an infected fly and enter the conjunctival sac of the host eye and mature into the adult stage in 1 month after 2 molts. Both adult and larval stages may live in conjunctival or lacrimal apparatus in the definitive host and cause conjunctivitis, keratitis, lacrimation, and ocular discharge. The adult females release first-stage larvae into the lacrimal secretions by ovoviviparity [7, 8]. Thus, the life cycle continues.

Clinical manifestations of thelaziasis range from ocular pruritus, lacrimation, epiphora, exudative conjunctivitis, or corneal edema to keratitis and corneal ulceration in severe cases leading to blindness [9]. Clinical diagnosis of thelaziasis in animals and humans may be difficult and misleading. A confirmed diagnosis of thelaziasis is usually made by the ophthalmologists based on visualization of the parasite on the conjunctiva. The eggs or larvae can be seen when tears or other eye secretions are examined under a microscope. Morphological differentiation between *T callipaeda* and *T californiensis* is based on the numbers of pre- and postcloacal papillae in the male and the position of the vulva in the female [10]. In general, the male *T callipaeda* worm has 8–10 pairs of preanal papillae and 5 pairs of postanal papillae, whereas the male *T californiensis* worm has 6–7 pairs of preanal papillae and 3 pairs of postanal papillae [2]. Although many drugs have promising effect, mechanical removal of the parasite is the only definitive treatment for human ocular thelaziasis [1].

## CASE REPORT

**Figure 1.**
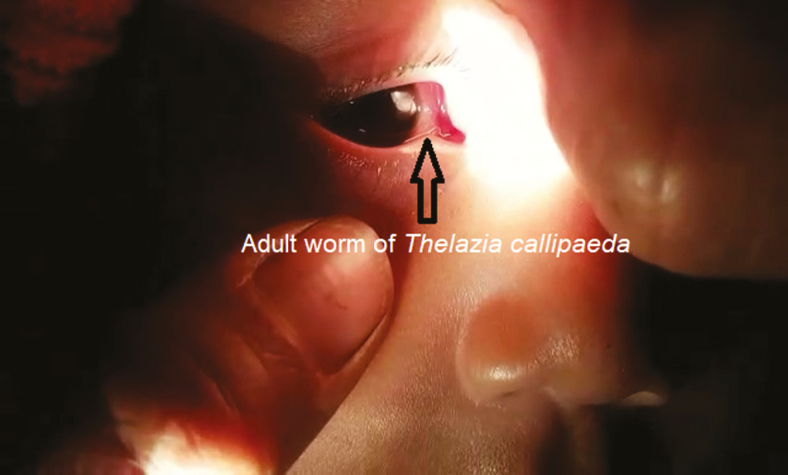
Adult worm of *Thelazia callipaeda* in the right eye of the child.

We report a rare case of human thelaziasis in a 6-month-old male child from the Rukum district (28^o^ 38’16”N, 84^o^ 27’17”E), Nepal. The district is at an altitude of 1581 meters above sea level, has a subtropical climate zone, an area of 2877 km^2^, and a population of 207290 (in 2011). The child is from a poor family, and he lives in a village where human and animals live in close proximity to each other. In this part of Nepal, people usually live in wooden houses where domestic cattle live on the ground floor and people live on the first floor. The patient was referred from a local eye hospital to an Ophthalmology clinic at Tribhuvan University Teaching Hospital, Kathmandu, Nepal. His mother noted a whitish, motile, thread-like worm in the lower fornix of the conjunctiva of the right eye (Figure 1). His mother also reported recent history of an insect (fly) sitting over the medial canthus of the right eye of the baby. Because the baby could not protect himself from the flies that sat on his eye, his mother reported that she came to the child, repeatedly leaving her household work, to wave the flies away from her baby’s eyes. On examination, it was noted that the child repeatedly rubbed his right eye. The patient was noted to have excessive lacrimation and conjunctival erythema and suffusion. No purulent discharge or trauma was noted. Visual acuity was within normal limits for age. Slit lamp examination was normal and did not demonstrate corneal abrasions, hypopyon, or retinal changes. A total of 6 worms were removed from the patient’s right eye. The worms were extracted from his right conjunctival sac by using a sterile cotton swab in our hospital, but 2 initial worms were removed by his mother with her bare finger. Ocular symptoms started to resolve rapidly after removal of the sixth worm. Of the 6 worms extracted, 4 were obtained for identification.

**Figure 2.**
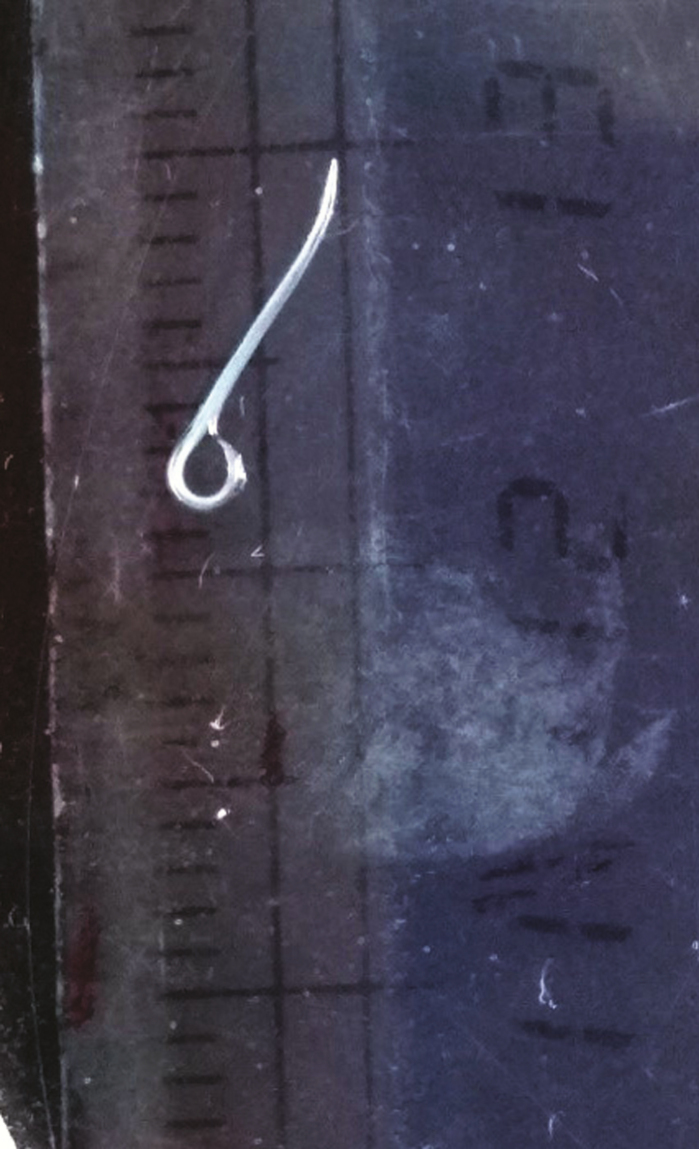
*Thelazia callipaeda* measuring approximately 10–12 mm.

**Figure 3.**
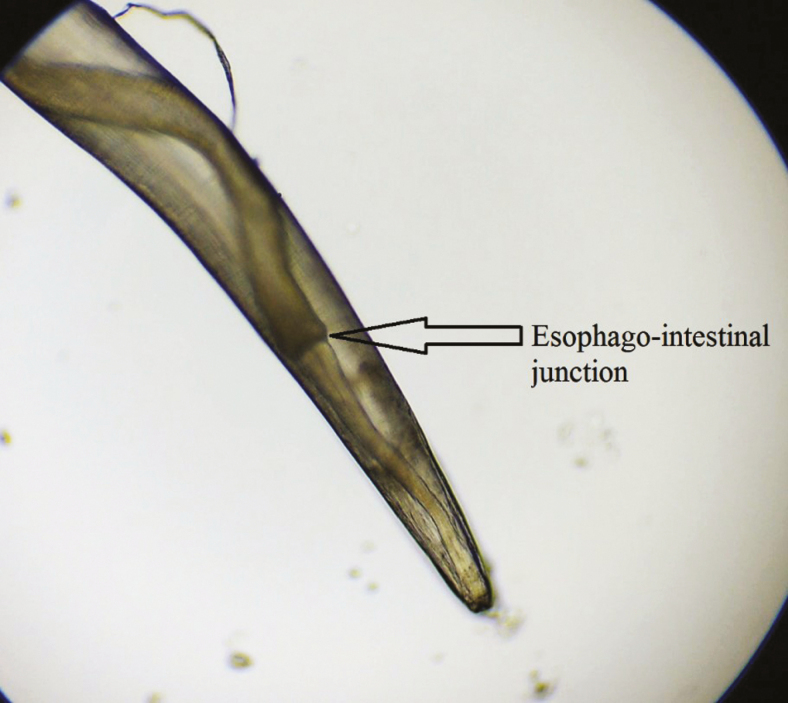
Anterior end of *Thelazia callipaeda* showing esophagointestinal junction.

**Figure 4.**
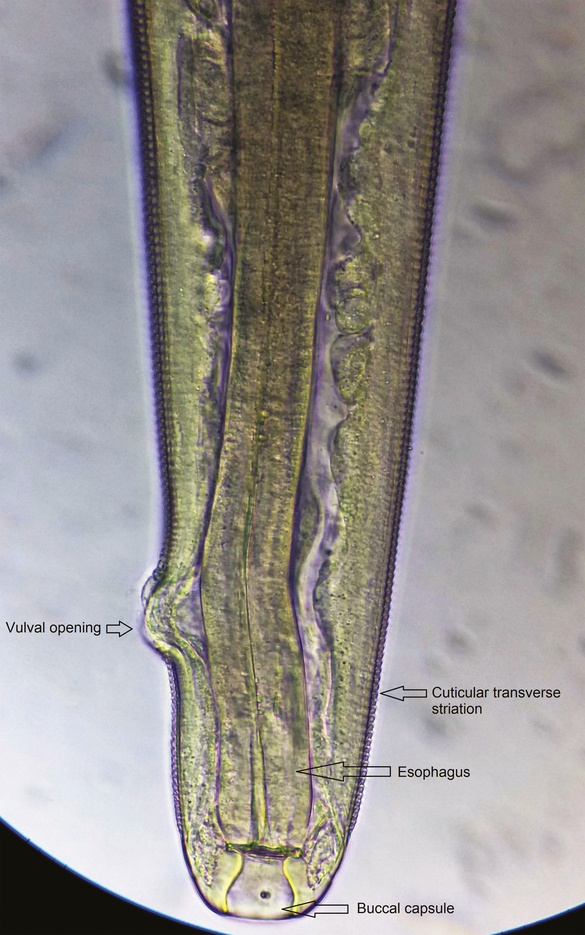
Anterior end of female *Thelazia callipaeda* showing buccal capsule, esophagus, vulval opening near to mouth and anterior to the esophageal-intestinal junction with cuticular transverse striations.

**Figure 5.**
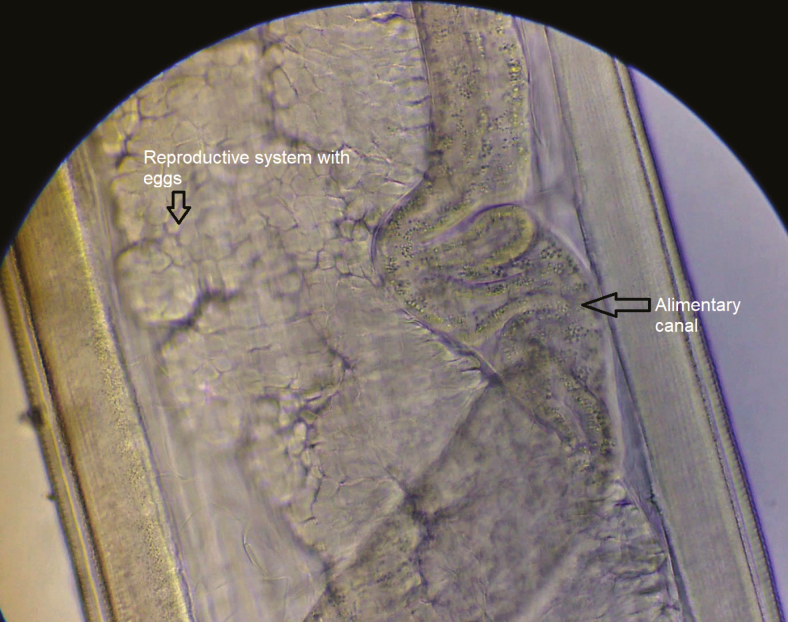
Middle portion of the worm showing the reproductive system with embryonated eggs and alimentary canal.

**Figure 6.**
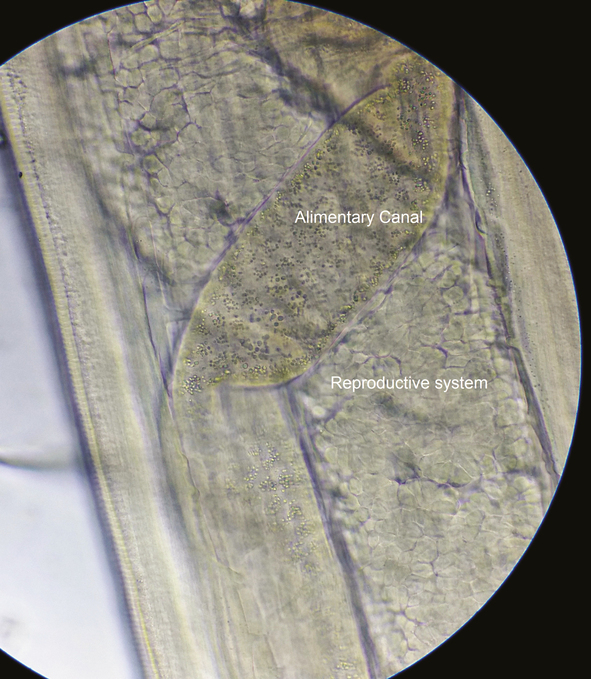
Middle portion of the worm showing the reproductive system with embryonated eggs and alimentary canal.

**Figure 7.**
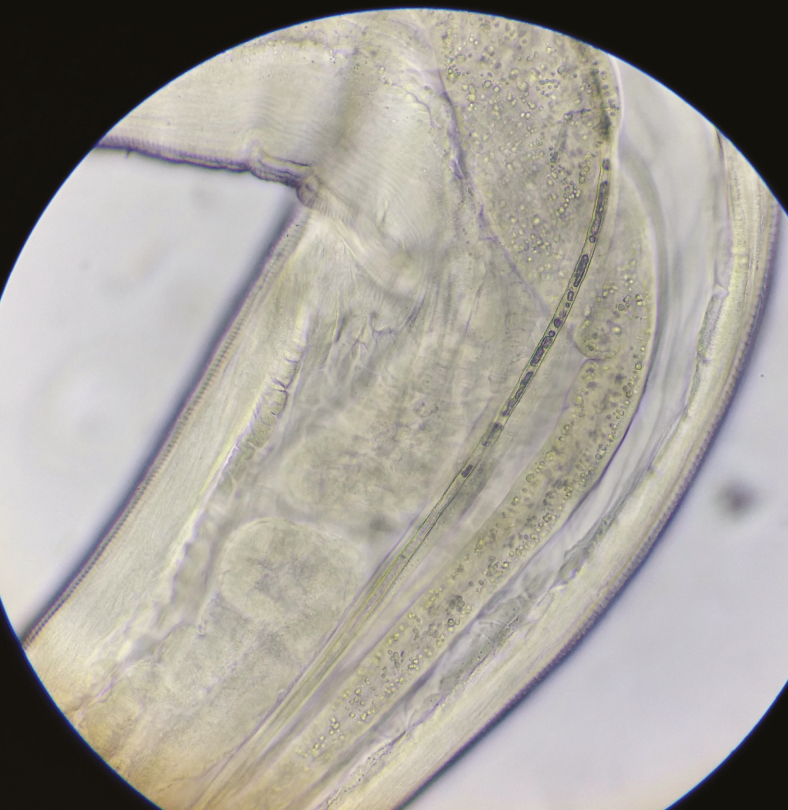
Distal end of *Thelazia callipaeda.*

**Figure 8.**
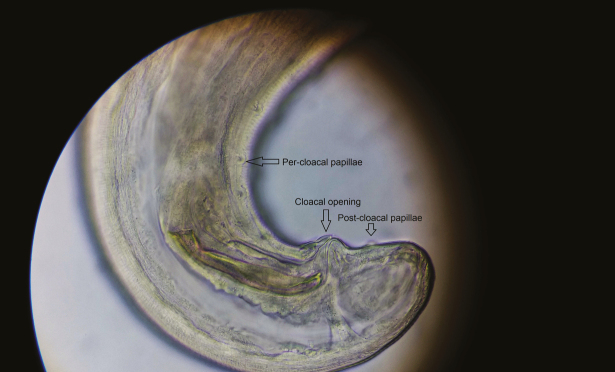
Caudal end of male *Thelazia callipaeda* showing characteristics curved tail end with shorter spicule and cloacal opening with pre- and postcloacal papillae.

All 4 worms were identified as *T callipaeda*, 1 was male, and 3 were female. The worms were white, thin thread-like, and measured approximately 10–12 mm in length (Figure 2) in female and 7–8 mm in male. The female worm had a serrated cuticle, buccal capsule, mouth opening with a hexagonal profile (Figures 3 and 4) with a long muscular esophagus (Figure 3) and a conical tail with the vulva opening located at the anterior portion of the esophagointestinal junction (Figures 3 and 4). The vulva opening was visible under high magnification of light microscope as a slight bulges part near the mouth with a smooth surface free of cuticles (Figure 4) while tracing laterally from the anterior part. The alimentary canal was well distinguished from the reproductive system, which contained embryonated eggs in the proximal uterus and first-stage larvae in the distal uterus (Figures 5–7). The anal opening of the worm was demonstrated at the caudal end. The male worm possessed similar buccal cavity but distinct esophageal-intestinal junction and characteristic curved tail end with shorter spicule with pre- and postcloacal papillae (Figure 8). Based on morphological keys, collected nematodes were identified as *T callipaeda*. For further identification, the microscopic photographic evidence was forwarded to the Centers for Disease Control and Prevention (Atlanta, GA) and confirmed as *T callipaeda*, based on morphological keys.

The patient was admitted for observation for 3 days, and worms were not observed on serial ocular examinations. He was discharged with tobramycin ophthalmic drop to prevent secondary bacterial infection. Patient followed up occurred in 2 weeks. On follow-up examination, conjunctival congestion was resolved and ophthalmoscopic examination was within normal limits. No worms were observed during this examination.

## DISCUSSION

To our knowledge, this is the first case report of human thelaziasis from Nepal, although many human cases have been reported from neighboring countries such as India and China. Ocular infections pose a critical threat internationally. The exposure of the eye directly to the environment renders it vulnerable to infrequent, but potentially devastating, infectious diseases caused by vector-borne parasites. Host defenses, once anatomical barriers are breached, may be ineffective in preventing complications including inflammation or loss of vision. Consequently, timely identification and treatment are critical to direct treatment and to prevent complications. Thelaziasis, like other ocular parasitic infections, occur more commonly in low socioeconomic regions and rural communities where humans live in close proximity with animals [12]. Unhygienic environments and animal waste attract a variety of flies near the house, similar to our case. Newborns and young children, as noted in our case report, are at risk due to increased risk of exposure and because they are unable to protect themselves from the flies sitting on their eyes.

Thelaziasis is a zoonotic disease caused by the *Thelazia* species and it affects the conjunctival sac, lacrimal duct, and lacrimal gland. Although our case presented early because it was detected by the patient‘s mother, late or neglected presentation may lead to sever diseases such as corneal ulceration and blindness [9]. The 2 species known to cause human thelaziasis are *T callipaeda* and *T californiensis. Thelazia callipaeda* is commonly found in Asia, whereas *T californiensis* is commonly found in the United States [11]. In a recent study, a third species, *Thelazia gulosa*, has been reported to cause human thelaziasis in the United States [12]. *Thelazia callipaeda* was first documented by Railliet and Henry in 1910 [2]. The only confirmed vector for *T callipaeda* is *P variegata* (Diptera, Drosophilidae, Steganinae) [4]. However, *Amiota okadai* is also considered to be a vector of this parasite in China (summarized by Otranto et al [13]). *Phortica* flies are attracted by the eye secretions of humans, animals (such as dogs), and carnivores, and they feed on these secretions. Due to their zoophilic feeding habit, *Phortica* flies act as a potential vector of *T callipaeda* [13]. The presence of *P variegata* is known in counties such as Buzau, Giurgiu, Constana, Caras-Severin, Mehendinti, Timis, Maramaures, Ialomita, and Teleorman [14]. Similar climatic and ecological conditions are found in some parts of Nepal. Thus, the vector is also probably present there. However, further entomological surveys are required for its confirmation. Thelaziasis has been reported in Japan, China, Southeast Asia, Burma, Indian subcontinent, Italy, France, Switzerland, Germany, Spain, Portugal, Bosnia, Croatia, Romania, and some parts of the United States [14]. However, it has not been reported in Nepal. Human thelaziasis is rare in infants, and our case is suggestive of prevalence of infection in Nepal and warrants further investigation.

It has been reported that treatment for canine infection of *T callipaeda* with topical organophosphates, 1% moxidectin, or a formulation containing 10% imidacloprid and 2.5% moxidectin is effective [1]. However, mechanical removal of parasites in humans remains the only curative option [1]. Nevertheless, due to the localization of the nematode, thelaziasis can be treated by direct application of drugs into the eyes. Patients with an intraocular infestation with *T callipaeda* have been successfully treated with a pars plana vitrectomy [10]. Michalski (1976) found that 2 mL of levamisole injected into the subconjunctival sac was more effective than levamisole given orally [10]. In case of *T californiensis*, removal of the worm will resolve the symptoms, and irrigation with Lugol’s iodine or 2%–3% boric acid is recommended immediately after worm removal or for the parasites that are in the lacrimal ducts where they cannot be removed manually [10]. Levamisole, either orally at 5 mg/kg or 2 mL injection into the conjunctival sac, has been recommended before the availability of ivermectin [12]. A dose of 2 mg/kg ivermectin given subcutaneously has also been shown to cure similar infestations in Asia and Europe [10, 12]. Because our case is an infant and manual removal of worms with a cotton swab or forceps was sufficient [12], levamisole and ivermectin were not used. However, the patient was under continuous observation by the mother, and an ophthalmologist performed frequent examinations. As soon as the worm was seen it was removed with the cotton swab. In additiona, tobramycin ophthalmic drops were given to the patient to prevent a secondary bacterial infection because the initial 2 worms were removed by bare finger. Currently, there is no vaccine for thelaziasis [10]. Thus, prevention of human thelaziasis should include stringent control of the fly vector, including use of bed nets to protect young children while they are sleeping and maintaining eye hygiene [1].

## CONCLUSIONS

Thelaziasis is an emerging zoonotic disease in low socioeconomic regions. It occurs in rural communities where humans live in close proximity with animals, similar to our case. Although this is the first reported case of human thelaziasis in Nepal, this condition is likely underreported, and a detailed epidemiological study on this parasite is needed to shed more light on present status of this infection in Nepal. Public health programs that promote hygienic environments free from insect vectors, such as flies, are vital to prevent ocular infections. In infected patients, removal of the worm may provide definitive treatment and prevent complications. Awareness of this emerging infection is critical for timely diagnosis among ophthalmologists and clinicians to prevent further devastating ocular complications such as blindness, especially in infants who cannot protect themselves from flies. Public health programs and prevention strategies in rural areas with an increased incidence of thelaziasis should include aggressive fly population management, netting around beds with eye hygiene, and treatment of infected domestic animals.
